# Does Local Area Social Mobility Affect Political Alienation?

**DOI:** 10.1177/00323217241283930

**Published:** 2024-10-11

**Authors:** Andrew McNeil, Patrick Sturgis

**Affiliations:** 1 Department of Political Science, University College London, London, UK; 2 Department of Methodology, London School of Economics, London, UK

**Keywords:** social mobility, political alienation, British politics, political geography

## Abstract

While existing research has considered how individual-level social mobility experiences affect a person’s political outlook, less attention has been paid to how historic levels of social mobility in local areas influence political attitudes and political behaviour. We link individual-level data from *the UK Household Longitudinal Study* to small area estimates of social class mobility derived from the decennial census. We find that living in a low absolute social mobility area was associated with a higher probability of voting ‘Leave’ in the 2016 UK European Union membership referendum. However, we find no evidence that historical social mobility rates in the local area predict abstention in general elections or attitudinal indicators of political alienation. Given declining rates of upwards mobility, and increasing levels of downwards mobility, our results have important implications for understanding geographies of political discontent.

## Introduction

It has been widely contended that a ‘geography of discontent’ (McCann, 2020) has pushed people living in ‘places that do not matter’ (Rodríguez-Pose, 2018) to vent their frustration by voting against the political mainstream. This has been driven by the fact that in most advanced democracies there are high levels of economic inequality, with recent research documenting a spatial divide in terms of equality of opportunity and outcome, that is, variation in social mobility across local areas (Bell et al., 2022; Buscha et al., 2021; Chetty et al., 2014). In modern democracies, the implicit social contract has been founded on the idea that individuals succeed through upward social mobility (Iversen and Soskice, 2019; Shafik, 2021). In short, we expect to do better than our parents and for our children to surpass our own achievements, or at least to fare no worse. This has become the dominant political discourse around fairness and equality (Ingram and Gamsu, 2022; Payne, 2017), and for most of the 20th century, it was fulfilled for most citizens (Buscha and Sturgis, 2018). However, fewer individuals, particularly men, are now upwardly mobile, with higher levels of downward and lower levels of upward mobility increasingly characterising social mobility regimes in the UK and other open market economies (Bukodi et al., 2015; Buscha and Sturgis, 2018). The gradual demise of the ‘golden era’ of upward social mobility seems likely to accelerate in the future (Bukodi and Goldthorpe, 2022), threatening to further breach the social contract, with potentially serious implications for the stability of political and economic systems.

We know from existing research that individual social mobility experiences shape political outlooks. Scholars have shown that an individual’s occupational trajectory is predictive of political attitudes, with evidence of both origin effects through socialisation, and to a lesser extent, the effect of the trajectory itself. Substantial origin effects have been observed for voting in the EU Referendum (McNeil and Haberstroh, 2023), and party choice in elections (Ares and van Ditmars, 2022; Clifford and Heath, 1993; De Graaf et al., 1995; McNeil, 2024), attitudes to redistribution (Jaime-Castillo, Marqués-Perales, 2019) and to immigrants (Paskov et al., 2020). Other studies have found effects of social mobility on attributions of success and failure, with the upwardly mobile attributing success to meritocratic conditions, while the downwardly mobile placing more weight on structural factors (Mijs et al., 2022).^1^ Downward educational mobility is also associated with higher levels of political distrust (Daenekindt et al., 2018), while Kurer and Van Staalduinen (2022) showed that people express political discontent through abstention and voting for radical parties when their intergenerational experience does not meet their *ex ante* expectations.

In this article, our focus is not on individual mobility experiences but on the historical level of social mobility in the local areas in which citizens currently reside. Individuals can observe mobility trajectories of work colleagues, neighbours, family, school friends and so on, and can use this information to draw inferences about the mobility chances in their locale (Reeves and Gimpel, 2012). In other words, an individual’s social networks are informative about local aggregate mobility experiences, and this enables them to form judgements about whether or not they live in an area where people can ‘get on in life’. This provides a context and reference point to assess how society functions regarding the implicit social contract. The implication is that living in an area characterised by low social mobility will engender political discontent and alienation, over and above one’s own mobility experience.

We test our expectations regarding low social mobility and political alienation using spatially granular estimates of social mobility from the UK decennial census (Buscha et al., 2021) linked to the *UK Household Longitudinal Survey* (*UKHLS*) at the level of Local Authority Districts (LADs). We measure the effects of local social mobility on political discontent using three outcome measures: voting Leave in the 2016 EU Referendum, political abstention (non-voting) in the 2010 General Election and political efficacy attitudes. We consider abstention in the 2010 General Election because this was the primary means of expressing disaffection with the political status quo at that time, while voting Leave provided an opportunity to express political alienation on the ballot paper. Finally, we consider political efficacy as a more direct attitudinal measure of political discontent.

A novel contribution of our article is that we assess the effect of both absolute and relative measures of social mobility on political attitudes and behaviour. This is an important distinction in the sociological literature on intergenerational mobility, yet no existing study has assessed whether and how they affect individual-level outcomes. The closest to this is Paskov et al. (2020) who analyse the effect of both absolute and relative mobility on a country-level average of individuals’ immigration attitudes.

The remainder of the article proceeds as follows. In the next section, we describe recent trends in social mobility in Britain and set out an account of how social mobility forms part of the implicit social contract. We then explain the mechanisms through which levels of local area social mobility affect political discontent and alienation. Next, we describe the data and measures to be used in our analysis before explaining our empirical strategy. We then detail our findings, and the discussion section considers some limitations of our research design and the implications of our findings for understanding how social mobility affects political attitudes and behaviour.

## Relevant literature

Over the past 40 years, political parties in the UK moved away from fierce social class-based competition (Dalton, 2002; Lipset and Rokkan, 1967; Pakulski and Waters, 1996), towards a growing consensus that a fair society should be based on promoting aspiration and *equality of opportunity*. This aspirational ‘promise’, or implicit social contract, is one where all citizens should be able to prosper irrespective of the material conditions of their origins (Iversen and Soskice, 2019). Parties from across the political spectrum have hailed education as the ‘great leveller’ in this regard, with the aim of equalising opportunities to move up the socio-economic ladder (Andersson, 2010). A political consensus developed in the later decades of the 20th Century based on the notion of meritocracy (Shafik, 2021); a ‘fair’ society is one that enables and promotes social mobility between generations, rather than aiming to achieve equality of outcome (Payne, 2017; Snee and Devine, 2018).

The transition from manufacturing and agrarian to service economies in most advanced democracies meant that the majority of citizens were upwardly mobile or stable (maintaining the social class position of their parents) in the middle and later decades of the 20th century. This was the so-called ‘golden era’ of upward social mobility when more ‘room at the top’ produced generations for whom the modal experience was to end up in a higher social class than the one they were raised in (Goldthorpe, 2016). As an example, for people in England and Wales born in the late 1960s, over 40% ended up in a higher social class group than their parents, with a further third being immobile (Buscha and Sturgis, 2018).^2^ Here we are referring to *absolute* mobility, the simple difference between an individual’s social class position and that of their parents, expressed in terms of the percentage who are upward, downward, or immobile across generations. Because absolute rates of mobility are affected by changes in the occupational structure of an economy over time, social mobility scholars also use *relative* measures (referred to as ‘social fluidity’) of intergenerational mobility. Relative mobility indicators adjust for changes in the size of social class groups, to yield measures of the risk of upward or downward mobility relative to other origin social classes. They are generally expressed as odds ratios and are considered to be a better indicator than absolute rates of the equality or ‘fairness’ of the mobility structure of a society or time period (Erikson and Goldthorpe, 1992). For example, although 38% of the late 1980s cohort in England and Wales were upwardly mobile, the odds of an individual from that generation born into the highest social class group being in that class as an adult were 20 times higher than someone born into the lowest social class group (Buscha and Sturgis, 2018).

Despite a widespread belief among politicians and commentators that social mobility has ‘ground to a halt’, or even gone into reverse, absolute social class mobility in Britain has actually remained more or less stable across cohorts since World War II (Bukodi et al., 2015; Buscha and Sturgis, 2018). Evidence from the census and birth cohort studies also indicates essentially stable, or slightly increasing, relative mobility from the 1950s to the early 1980s (Bukodi et al., 2015; Buscha and Sturgis, 2018). The UK appears to have at least as much, if not more, total mobility and is as fluid as its European peers (Bukodi et al., 2019). Thus, we see the UK as an interesting case given the ‘aspirational’ social contract, but it is by no means unique in terms of its social mobility regime.

Upwardly mobile individuals generally credit their own hard work for their successes (Kluegel and Smith, 1986), though they may also believe the political and market institutions provided a platform; they succeeded according to the implicit social contract. By contrast, for the many who were downwardly mobile or remained in the same ‘low’ class position as their parents, the promise of the aspirational society failed to materialise. Kurer and Van Staalduinen (2022) showed how individuals who are disappointed with their adult outcomes relative to their expectations are more likely to abstain from voting and more likely to vote for radical parties. This effect is intensified as individuals attribute personal welfare gains and losses in an asymmetric manner, placing more responsibility on the government if changes are negative (Larsen, 2021).

While the national context is clearly important, more relevant for our purposes here is social mobility at a local level. This focus on the local level aligns with the recent turn in social mobility research towards understanding the distribution of social mobility at small spatial scales, notably in the work of Raj Chetty and colleagues in the US (Chetty et al., 2014). Chetty et al. (2016) showed that the strength of the link between social origins and adult outcomes is highly dependent on the characteristics of the local area an individual grew up in. In the UK, large differences in both absolute and relative mobility have been found between regions (Bell et al., 2022; Friedman and Macmillan, 2017). Using the ONS longitudinal study, Buscha et al. (2021) found substantial variability in social class mobility between LADs in England and Wales, covering the period 1971 to 2011. This heterogeneity was evident across the whole country, with every major region containing local authorities in the top and bottom deciles of the social mobility distribution, belying the conventional characterisation of a simple ‘north-south divide’ in social mobility chances.

Our objective in this article is to assess whether this heterogeneity in local-level social mobility influenced individuals’ propensity to express attitudes and behaviours characteristic of political alienation. If people see a greater proportion of their local area social network ‘succeed’ according to the implicit social contract, they will be more likely to support the political status quo. Conversely, if people see few examples of upwards mobility in their local area, their support of the status quo ‘aspirational’ society is likely to diminish, and political disappointment set in. While it is unlikely that individuals will have any familiarity with social mobility statistics, we argue that lived experiences can provide a sufficient heuristic to derive an approximately accurate assessment of local-level social mobility. This mechanism of direct personal experience also leads us to expect that absolute mobility will be more influential on political alienation than will social fluidity. This is because, as noted above, absolute mobility can be observed directly via the experiences of an individual’s wider social network (Breen, 1987; Buscha and Sturgis, 2018; Hout and Hauser, 1992). Relative mobility, on the other hand, is more difficult to observe as it requires an assessment, not only of levels of upward and downward mobility, but of how these mobility chances are distributed across social class origin states.

## Theoretical expectations

We expect political discontent and alienation arising from low social mobility in the local area to manifest in both political attitudes and voting behaviour. We are aware of only one other article that tests the effect of local social mobility on political behaviour, and their focus is on political abstention only (Kim et al., 2021). These authors show that people living in US counties with lower-income mobility were less likely to vote in elections. This is consistent with our theoretical expectation in this article.

More broadly, we link to a literature that argues that living in a ‘left-behind’ area, or ‘place that does not matter’ (Rodríguez-Pose, 2018), creates a ‘geography of discontent’ (McCann, 2020). Lower house prices (Adler and Ansell, 2020), greater exposure to austerity (Fetzer, 2019), economic stagnation (Rodríguez-Pose et al., 2023) or vulnerability to the Chinese import shock (Colantone and Stanig, 2018) are just some of the explanations offered as place-based mechanisms. Our expectations for spatial contextual-level effects of mobility are in part driven by the effects of individual-level social mobility on attitudes and political support (e.g. Daenekindt et al., 2018; McNeil and Haberstroh, 2023), and more widely how relative status decline (Gest et al., 2018; Gidron and Hall, 2017) and disappointed expectations (Kurer and Van Staalduinen, 2022) affects abstention and radical political party support, particularly when radical right parties are able to associate blame for status decline with outgroups (Gidron and Hall, 2017; Sobolewska and Ford, 2020). The connection between these predictors and our articulation of a social contract based on mobility is an idea of societal fairness – and we view these explanations as being in parallel rather than in competition.

We follow Kurer and van Staalduinen (2022) as to how political discontent manifests. Given the dissatisfaction with mainstream politics, and the lack of alternatives with any realistic chance of making it into government, we expect that this will be reflected through political abstention. This is particularly pertinent in the 2010 General Election which preceded the rise of the UK Independence Party (UKIP)^3^ and the prospect for a referendum regarding the UK’s membership of the European Union. The Conservative Party’s manifesto for the 2015 General Election included a commitment to hold the EU Referendum, which may have incentivised individuals to turnout to later place an anti-establishment ‘Leave’ vote. We concentrate on the 2010 General Election for this reason. For some, the European Union membership referendum offered an alternative to abstention to express political discontent, for others who previously voted in the 2010 General Election, there is a clear choice between the status quo, ‘Remain’, supported by the mainstream parties, and the alternative, ‘Leave’. The EU Referendum may have been a ‘unique opportunity’ for those who perceived ‘The Labour Party abandonment of the working class’ and ‘their lack of control over the nation’s political economy’ (Telford and Wistow, 2020: 556 and 568). The EU Referendum attracted many voters who did not vote in previous General Elections, 60% of whom voted to ‘Leave’ (Swales, 2016).^4^ Citizens’ attitudes towards political efficacy may be one of the underlying psychological reasons mediating these voting behaviours. Thus, we view it as a direct attitudinal measure of political discontent. We now outline these three measures, and our expectations, in more detail.

First, we expect lower social mobility areas to foster abstention in UK General Elections, in line with the findings of Kim et al. (2021) in the US. The single-member district electoral system in the UK lends itself to fewer parties (Cox, 1997; Duverger, 1969). The two major parties in the UK, the Conservatives and Labour, converged on major political issues over the 1990s and 2000s. Tony Blair explicitly aimed at the aspirational electorate which was captured by Margaret Thatcher in the 1980s, signing up to much of her agenda (Iversen and Soskice, 2019). Given the lack of an alternative, capable of forming a government to this ‘cartel’ party politics (Katz and Mair, 1995), for those dissatisfied with the mainstream political paradigm, an alternative was abstention. While this is still true in countries with proportional electoral systems, these systems allow individuals to express their disappointment, economic or otherwise, through supporting anti-system parties (Hopkin, 2020). In the UK majoritarian system, when anti-system parties, such as UKIP, emerge they struggle to convert their support into electoral success. For example, even in the 2015 General Election, a year prior to the EU referendum, UKIP won only a single seat despite having a vote share of 13%. This leads to our first hypothesis:

*Hypothesis 1 (H1). People living in areas with lower levels of absolute upward social mobility/relative mobility were more likely to abstain in the 2010 General Election.*

Second, we expect low local area social mobility to increase the probability of voting ‘Leave’ in the EU Referendum held on 23 June 2016. The literature on the characteristics of Leave voters, and anti-system parties more generally, has identified a wide range of individual characteristics: lower educational attainment (Alabrese et al., 2019; Hobolt, 2016), occupational status (Evans and Tilley, 2017), subjective social status (Gidron and Hall, 2017), and being less post-materialistic (Norris and Inglehart, 2019).^5^ Beyond attributes at the individual level, studies have identified socio-tropic effects whereby living in a ‘left-behind’ area, or ‘place that does not matter’ (Rodríguez-Pose, 2018), creates a ‘geography of discontent’ (McCann, 2020). The broad logic of individual and place-based explanations is similar. They rely on individuals being dissatisfied with their own lot or the fortune of the place in which they live, and these factors influence and reinforce each other. Voting ‘Leave’ in the EU Referendum was seen by many as a way to voice disappointment with the political mainstream and the social contract it represented. Our second hypothesis is therefore:

*Hypothesis 2 (H2). People living in areas with lower levels of absolute upward social mobility/relative mobility were more likely to vote ‘Leave’ in the 2016 EU referendum.*

Third, we expect local social mobility to affect individuals’ sense of political efficacy, that is, beliefs about the perceived (in)adequacy of politics, politicians and their (in)ability to influence political outcomes (Jennings et al., 2016). Indeed, these kinds of political attitudes may mediate any link between area-level characteristics and voting behaviour. As Fox (2021: 19) notes, “acts such as refusing to vote, rioting, or voting for Brexit are not indicators of political alienation in themselves, but rather are caused by the attitudes that reflect alienation”. Here, we expect that living in areas with low social mobility will be associated with lower political efficacy, as the promise of the social contract will be perceived to have disappointed these locales. This leads to our third hypothesis:

*Hypothesis 3 (H3). People living in areas with lower levels of absolute upward social mobility/relative mobility will express lower levels of political efficacy.*

## Data and measures

Individual-level survey data are drawn from *the UKHLS* (University Of Essex, 2022), a household panel survey with annual waves running from 2009, with interviews conducted with all adult household members (16+) in approximately 40,000 households at wave 1. Data collection is through interviews in the respondents’ homes or through online self-completion. In Wave 8, interviews were conducted with 37,565 respondents representing a wave response rate of 87% (based on responses from those who responded in the previous round).^6^ *UKHLS* incorporates new members where an original sample household member moves to a new household, and they form new households. Our estimates are weighted to account for unequal selection probabilities in the sample design, nonresponse at wave 1 and attrition across waves.

Our key independent variables of area-level social mobility are taken from the analyses reported in Buscha et al. (2021). These authors produce estimates of absolute and relative mobility at the LAD using the *Office of National Statistics Longitudinal Study*. This is a 1% sample of the decennial Censuses from 1971 to 2011 in England and Wales, with approximately 500,000 individual records linked at each census year (Shelton et al., 2019). The estimates we use here are based on pooled data from across the 1971 to 2011 censuses, so these variables represent the historical experience of social mobility over the three decades prior to the survey wave. Mobility estimates are produced by comparing study members’ social class in adulthood with the class positions of their parents 20 years earlier, when study members were aged between 8 and 18 years. The measure of social class used is the National Statistics Socio-Economic Classification (NS-SEC), which measures social class via occupation, size of organisation, and managerial responsibilities (Rose et al., 2005). We define LAD-level absolute upwards mobility as the percentage of individuals who moved from NS-SEC groups 4–8 origin class (‘low’ origin class) to NS-SEC groups 1–3 destination class (‘high’ destination class).^7^ The relative mobility measure is the ratio of the odds of a high origin individual ending up in a high rather than a low destination class, to the odds of a low origin individual ending up in a high rather than a low destination class. Note that this means higher values indicate more social mobility for the absolute measure but lower levels of relative mobility. We have replicated these analyses using a measure of ‘long-range’ social mobility, where upwards mobility is defined as moving from the bottom two to the top three classes and the results are substantively unchanged (Supplementary Table S2).

**Figure 1.**
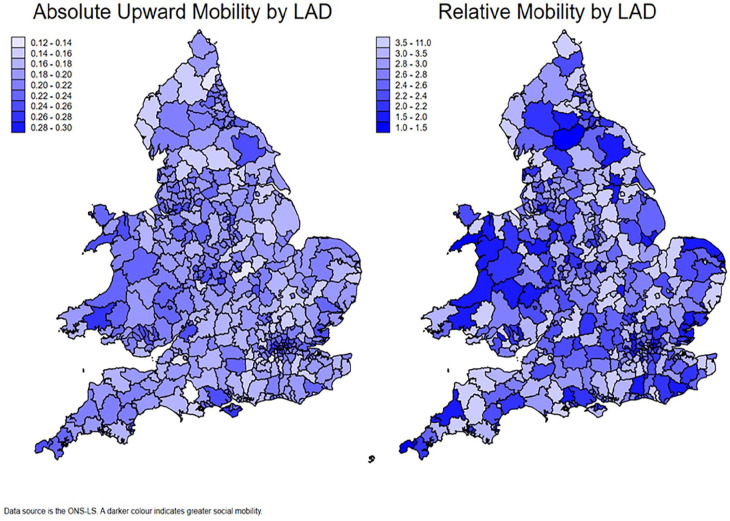
Absolute and relative mobility across Local Authority Districts in England and Wales.

Given our area-level social mobility estimates are based on 1991 LAD boundaries and the data from *UKHLS* uses current boundaries, we match our estimates using LAD area proportions. For example, if a current LAD boundary has 80% of its area from a 1991 LAD and 20% from another, we would use the weighted average estimate of social mobility from the two 1991 LAD boundaries. While this boundary matching is not perfect, less than 10% of current LADs shared a lower than 90% match with a 1991 LAD boundary. Our models contain data from up to 330 LADs. Figure 1 shows maps of absolute and relative social mobility across the 335 LADs in England and Wales, revealing the high degree of heterogeneity across regions and local authorities. Correlations between mobility estimates, absolute and relative, and the LAD-level covariates are reported in Supplementary Table S3.

The first dependent variable is whether an individual turned out to vote in the 2010 General Election, which was held on 6^th^ May. We code voted as (0) and not voted as (1). We exclude ‘refusals’, ‘don’t knows’ and ‘can’t votes’ from our analysis. The voting question was only administered to respondents who were interviewed in 2010 after the General Election had taken place (36% of the total sample for that wave). We models for the 2010 election as this was the most proximal to the period covered by the social mobility estimates, and other General Elections (2015 and 2017) available in the UKHLS included Brexit as a salient issue. Models for the 2015 and 2017 elections are available in Supplementary Tables S4 and S5, as is a version which uses a variable from different waves which elicited turnout intention for the next General Election (Supplementary Tables S6–S8).

Our second dependent variable is voting in the EU Referendum, using the Wave 8 question, ‘*Should the United Kingdom remain a member of the European Union or leave the European Union?*’. We code ‘Remain’ as (0) and ‘Leave’ as (1). Note that the *UKHLS* has an underrepresentation of ‘Leave’ voters in the sample, 45%, compared to the actual result of 51.9%.

For the attitudinal indicators, we fitted an exploratory factor analysis of four items on political efficacy where the response scales have 5 points running from strongly agree to strongly disagree:

1. I consider myself to be well qualified to participate in politics.
2. I think I am better informed about politics than most people.
3. Public officials do not care much about what people like me think.
4. People like me do not have any say in what the government does.

This produced a two factor solution with the first two questions loading on Factor 1 and questions 3 and 4 loading on Factor 2. We interpret Factor 1 as capability to understand and participate in political processes, while Factor 2 speaks to feeling of the government’s closeness and responsiveness to ‘people like them’. We take the predicted value for each factor as our measures of political efficacy. Eigenvalues and factor loadings are available in Supplementary Tables S19 and S20.

To measure individual mobility trajectories, we code respondents to four groups based on their origin and destination social class: 1. ‘Immobile high’ (respondent and parents are both NS-SEC 1–3); 2. ‘Immobile low’ (respondent and parents are NS-SEC 4–8); 3. ‘Downwardly mobile’ (respondent is NS-SEC 4–8 and parents NS-SEC 1–3); and 4. ‘Upwardly mobile’ (respondent is NS-SEC 1–3 and parents are NS-SEC 4–8). The parental measure is calculated using the dominance approach, that is, the highest of either mother or father, including cases where occupational status is available for just one parent. Only respondents aged 30 years or older are included because most people do not reach occupational maturity before this age and so the mobility trajectory cannot be observed. Using NS-SEC as the measure of origin and destination class also means those out of employment and those without information for parental occupation are excluded.

Social mobility is correlated with other causes of Brexit voting, so we control for potentially confounding variables in our model: sex, age, educational attainment, ethnicity, marital status, long-standing illness and income. We include a dummy indicator of whether the respondent was interviewed prior to or after the EU referendum on 23 June 2016. This is because the interviews for Wave 8 were conducted over 24 months spanning 2016 and 2017 so some individuals may have altered their response after the result was known.^8^ We also include a set of area-level controls: the percentage of the population with a degree, percentage who are not British (by citizenship), the median age, percentage who are white, percentage unemployed, the GVA of the area, and the change between 2004 and 2016 in the percentage of individuals who are British born. These are drawn from the 2011 Census and the Office of National Statistics.^9,10^

## Empirical strategy

Due to the hierarchical structure of our data, with individuals nested in LADs, we use hierarchical (multilevel) models (Goldstein, 1991). We use a logistic link-function for the abstention and EU referendum vote where the outcomes are dichotomous, but the results are substantively unchanged using a linear probability model (see Tables 9 and 10 in the Supplementary Materials). Models are fitted in stages for each of the three dependent variables. Model 1 is the ‘empty’ model containing no covariates, Model 2 includes LAD-level absolute mobility only, Model 3 only LAD-level relative mobility, Model 4 both LAD absolute and relative mobility, and finally, Model 5 includes both measures of LAD mobility and the individual and LAD-level covariates.^11^ We also include a version of the model in the Supplementary Materials with only individual-level controls and only LAD-level controls, respectively, the findings are substantively the same (Supplementary Tables S17 and S18).

The model has the following form (expressed here in its linear form for simplicity):

(1) $$y_{ij}=\beta_{0}+\beta_{Abs}x_{Abs\;j}+\beta_{Rel}x_{{Rel}_{j}}+\beta^{\prime }{x^{\prime }}_{ij}+\beta^{"}x{"}_{j}+\mu_{j}+e_{ij}$$

where *y* is the outcome of interest for individual *i*, in LAD *j*, $x_{Abs\;j}$ is the LAD-level absolute mobility, LAD-level relative mobility is $x_{Rel\;j}$ , ${x^{\prime }}_{ij}$ and $x{"}_{j}$ are the vectors of individual- and LAD-level predictors, respectively, and $\beta_{0}$ is an intercept that is allowed to vary between LADs through the random effect $\mu_{j}$ , which is assumed to have normally distributed variance $\sigma_{u}^{2}$ . $\beta_{Abs}$ and $\beta_{Rel}$ are the key parameters of interest to be estimated, denoting the expected change in the outcome *y* for unit changes in absolute and relative mobility, respectively.

The sample size for the empty ‘Brexit’ model (Model 6, Table 2) is 25,635, which is reduced to 21,095 with the restriction to respondents who are aged 30 or above. Dropping respondents resident in Scotland and Northern Ireland reduces this further to 17,226, while including the respondent’s own mobility trajectory and covariates yields a sample size of 8,503. Because the question on turnout in the 2010 General Election was only asked to a subset of respondents, the sample size for these models is 5,604. For comparability, we use the most restrictive (smallest) sample across all five versions of the model. Results for models which use the maximum sample size for each specification are included in Supplementary Tables S11–S13, and show a substantively unchanged pattern of findings.

## Results

Turning to the 2010 UK General Election turnout models, we first fit the ‘empty’ Model 1 (Table 1) to obtain the variance and intra-class correlation (ICC) across the 2 levels of the hierarchical structure. While most variability comes from the individual level, 9% of the variability in turnout is between LADs. In Models 2 and 3, we find small and statistically non-significant associations between both absolute and relative measures of mobility and abstention. The conditional ICC (i.e. after controlling for LAD absolute and relative mobility, respectively) barely changes – less than 0.03 percentage points. When we include the covariates, there is a weak association between absolute mobility and abstention – a 1 percentage point increase in LAD absolute mobility is associated with a 0.044 decrease in the log odds of abstention (i.e., a decrease in the odds by 4.3%), although this is not statistically significant [p = 0.11]. The coefficient for relative mobility is in the opposite direction to our expectation (more social fluidity is associated with a higher probability of voting) but this is also not significant (p = 0.18). For further interpretation, we plot predicted probabilities over the range of LAD mobility estimates in Supplementary Figure S3.

**Table 1.** Multilevel models predicting abstention in the 2010 General Election (log odds).

	(1)	(2)	(3)	(4)	(5)
	Empty	Absolute	Relative	Absolute and Relative	Absolute and Relative and Covariates
LAD absolute mobility		−0.00387		−0.0113	−0.0444
		(0.0193)		(0.0221)	(0.0274)
LAD relative mobility			−0.0429	−0.0642	−0.124
			(0.0777)	(0.0895)	(0.0915)
Individual social mobility (base always high)					
Downwards					0.492**
					(0.167)
Upwards					0.401**
					(0.123)
Always low					0.584***
					(0.145)
Individual-level controls	N	N	N	N	Y
LAD-level controls	N	N	N	N	Y
Observations	5,604	5,604	5,604	5,604	5,604
Number of groups	316	316	316	316	316
Random effects					
Variance(LAD)	0.325	0.324	0.325	0.322	0.23
	(0.0692)	(0.0679)	(0.0695)	(0.0679)	(0.0541)

Robust standard errors are in parentheses. LAD-level controls are: % with degree, % not British, median age, % white, % unemployed, GVA, change in non-British born between 2004 and 2016. Individual-level controls are age, sex, race, marital status, health and education. The full regression table is available in Supplementary Table S14. Individuals are coded as 0 (voted) or 1 (did not vote). Coefficients are log odds.

*** p < 0.001, **p < 0.01, *p < 0.05.

As existing studies have shown, an individual’s own social mobility trajectory is related to the tendency to abstain. The ‘immobile low’ are the most likely to abstain, followed by the downwardly mobile, then the upwardly mobile, and finally the ‘immobile high’. The odds of the ‘immobile low’ abstaining are 79% higher than the ‘immobile high’ in the 2010 General Election [p < 0.001]. These are included in the main tables for reference, full regression tables are available in Supplementary Table S14.

We repeat this analysis for the 2015 and 2017 UK General Elections in Supplementary Tables S4 and S5 – although as noted earlier these are not our preferred specifications because of distance in time from the mobility estimates and contamination from the EU Referendum. The results are similar to those in Table 1; living in a low absolute mobility LAD is associated with an increased probability of abstaining, albeit this is not statistically significant. Analysing vote intention for the next election, which includes a larger sample as respondents from all months were asked this question, shows that this pattern is again observed, but is only statistically significant in Wave 3 (2011–2012) (Supplementary Tables S6–S8).

Next, we repeat the same sequence of models for voting Leave in the EU Referendum. The variance components from the ‘empty’ Model 6, Table 2, shows that again most of the variation is from the individual level, but the LAD-level variance is now slightly greater, 9.5%. In Model 7, we find that an additional percentage point of LAD absolute mobility is associated with a decrease in the log odds of voting ‘Leave’ of 0.0431 [p = 0.007]. By contrast, the coefficient for relative mobility is not statistically significant (Model 8). The conditional ICC in Model 7 (absolute mobility) is 9.1% and in Model 8 (relative mobility) 9.5%. With both absolute and relative mobility in the model, but no other covariates, Model 9, the previous relationships are essentially unchanged, with the coefficient for absolute mobility now −0.0476 [p = 0.013]. When including individual and LAD-level covariates, Model 10, absolute mobility has a similar effect size as in the previous iterations of the models, an additional percentage point increase in LAD-level absolute upwards mobility is associated with a 0.0431 decrease in the log odds of an individual voting ‘Leave’ [p = 0.024]. This equates to a 6.8 percentage point difference in voting ‘Leave’ for an individual living in a low absolute mobility area (10th percentile) compared to a high absolute mobility area (90th percentile), when holding other covariates at their means. LAD relative mobility has no statistically significant effect on ‘Leave’ support in the full model, and the magnitude is substantively small in any event.

**Table 2.** Multilevel logistic models, LAD-level mobility and ‘Leave’ support (Remain = 0, Leave = 1, log odds).

	(6)	(7)	(8)	(9)	(10)
	Empty	Absolute	Relative	Both	Absolute and Relative and Covariates
LAD absolute mobility		−0.0431**		−0.0476*	−0.0431*
		(0.0160)		(0.0192)	(0.0191)
LAD relative mobility			0.0501	−0.0336	0.00843
			(0.0487)	(0.0604)	(0.0532)
Individual social mobility (base always high)					
Downwards					0.428***
					(0.0900)
Upwards					0.259**
					(0.0841)
Always low					0.730***
					(0.0847)
Individual-level controls	N	N	N	N	Y
LAD-level controls	N	N	N	N	Y
Observations	8,503	8,503	8,503	8,503	8,503
Number of groups	329	329	329	329	329
Random Effects					
var(LAD)	0.346	0.328	0.344	0.327	0.157
	(0.0493)	(0.0451)	(0.0488)	(0.0451)	(0.0381)

Robust standard errors are in parentheses. LAD-level controls are: % with degree, % not British, median age, % white, % unemployed, GVA, change in non-British born between 2004 and 2016. Individual-level controls are age, sex, race, marital status, health and education. 0 coded as ‘Remain’ and 1 as ‘Leave’. Coefficients are log odds. The full regression table is available in Supplementary Table S14.

*** p < 0.001, **p < 0.01, *p < 0.05.

Individual-level social mobility also matters for the probability of voting ‘Leave’. In line with previous findings (McNeil and Haberstroh, 2023), the ‘immobile low’ are the most likely to vote ‘Leave’ and the ‘immobile high’ the least likely. The upwardly mobile are less likely to vote ‘Leave’ than the downwardly mobile but both are in-between the two immobile groups. The difference between the immobile groups is large, especially given that we conditioned on educational attainment and income. The odds of the immobile low voting ‘Leave’ are 108% higher than for the immobile high.

Finally, we turn to the models for political efficacy attitudes, with the coefficient estimates presented in Table 3 (Factor 1) and Table 4 (Factor 2). In Models 15 and 20 which include covariates, both absolute and relative mobility coefficients are small in magnitude and not statistically significant. Prior to including the LAD and individual controls, we do find that low LAD absolute mobility is associated with lower levels of ‘capability to understand and participate in political processes’. The proportion of variance at the LAD level in the empty models of both Factor 1 and Factor (Models 11 and 16) is less than 4%.^12^ In addition, the magnitude of an individual’s own social mobility is smaller for political efficacy than our other two variables, although it remains statistically significant.

**Table 3.** Multilevel Models, LAD-level Mobility and Attitudes of Political Efficacy, First Factor (Capability to Understand and Participate in Political Processes).

	(11)	(12)	(13)	(14)	(15)
	Empty	Absolute	Relative	Absolute and Relative	Absolute and Relative and Covariates
LAD absolute mobility		−0.0116*		−0.0134**	−0.00397
		(0.00447)		(0.00492)	(0.00384)
LAD relative mobility			0.0105	−0.0138	−0.00197
			(0.0156)	(0.0160)	(0.0128)
Individual social mobility (base always high)					
Downwards					0.138***
					(0.0231)
Upwards					0.0620**
					(0.0192)
Always low					0.236***
					(0.0208)
Individual-level controls	N	N	N	N	Y
					
LAD-level controls	N	N	N	N	Y
Observations	13,334	13,334	13,334	13,334	13,334
Number of groups	330	330	330	330	330
Random effects					
Variance (LAD)	0.0236	0.0222	0.0235	0.0221	0.00654
	(0.0111)	(0.00320)	(0.00347)	(0.00320)	(0.00150)
Variance (individual)	0.591	0.591	0.591	0.591	0.486
	(0.00779)	(0.00779)	(0.00779)	(0.00779)	(0.00612)

Robust standard errors are in parentheses. The factor is rescaled to mean zero, s.d. = 0.79, minimum score is -2.04, and maximum 1.51. The scores should be interpreted as lower score is higher belief in political efficacy. LAD level controls are: % with degree, % not British, median age, % white, % unemployed, GVA, change in non-British born between 2004 and 2016. Individual level controls are: age, sex, race, marital status, health, education. Full regression table available in the Supplementary Material Table 14.

*** p < 0.001, **p < 0.01, *p < 0.05.

**Table 4.** Multilevel Models, LAD-level Mobility and Attitudes of Political Efficacy, Second Factor (Feeling of the Government’s Closeness and Responsiveness to ‘People Like Them’).

	(16)	(17)	(18)	(19)	(20)
	Empty	Absolute	Relative	Absolute and Relative	Absolute and Relative and Covariates
LAD absolute mobility		0.00544		0.00411	−0.00177
		(0.00408)		(0.00503)	(0.00404)
LAD relative mobility			−0.0174	−0.00998	−0.0104
			(0.0134)	(0.0173)	(0.0129)
Individual social mobility (base always high)					
Downwards					0.119***
					(0.0231)
Upwards					0.0724**
					(0.0221)
Always low					0.202***
					(0.0227)
Individual-level controls	N	N	N	N	Y
LAD-level controls	N	N	N	N	Y
Observations	13,334	13,334	13,334	13,334	13,334
Number of groups	330	330	330	330	330
Random effects					
Variance (LAD)	0.0206	0.0205	0.0205	0.0205	0.00851
	(0.00283)	(0.00285)	(0.00281)	(0.00283)	(0.00174)
Variance (individual)	0.568	0.568	0.568	0.568	0.536
	(0.00670)	(0.00670)	(0.00670)	(0.00670)	(0.00678)

Robust standard errors are in parentheses. The factor is rescaled to mean zero, s.d. = 0.76, minimum score is -2.13, and maximum 1.50. The scores should be interpreted as lower score is higher belief in political efficacy. LAD level controls are: % with degree, % not British, median age, % white, % unemployed, GVA, change in non-British born between 2004 and 2016. Individual level controls are: age, sex, race, marital status, health, education. Full regression table available in the Supplementary Material Table 14

*** p < 0.001, **p < 0.01, *p < 0.05.

We have treated the LAD-level absolute and relative mobility measures as continuous, which is potentially problematic for both methodological and substantive reasons. We therefore provide robustness checks in the Supplementary Materials in which we test the sensitivity of our conclusions to how we specify the distributions of these key variables. First, we exclude the bottom and top deciles of the distribution (Supplementary Table S15) to test the sensitivity of the results to extreme values. This is particularly relevant for relative mobility because there are a small number of LADs with very large odds ratios. Because these measures are estimates from sample data (see Buscha and Sturgis, 2018), such high values are likely, in part, to reflect sampling variability. In addition, we replicate our analyses using ‘long-range’ measures of relative mobility which treats upwards mobility as occurring from the bottom to the top of the NS-SEC scale (Supplementary Table S2). We also check the linearity assumption for the mobility measures by coding them to quartiles and including these as dummy variables (Supplementary Table S16).

Our estimates of area social mobility are taken as the current location of the respondent. However, it is possible that the mobility experiences in the area an individual grew up in are more consequential than where they currently reside. We are limited in the extent to which we can consider this, due to the lack of information in the survey on where respondents were living when they were growing up. We can partially address this though, by fitting models to the subset of ‘non-movers’, measured as those who currently live in the same place they did when they were born. Under this specification (see Supplementary Table S22), the coefficient for LAD absolute mobility in the ‘Leave’ support model is similar to the full sample model (−0.0405 when including individual and LAD covariates). While this is no longer statistically significant, this model is estimated with a smaller sample size (n = 3,229) and are therefore estimated with less precision. The area-level absolute mobility estimates in the political efficacy models, for both political efficacy dimensions, remain substantively small and non-significant under the alternative specifications. However, for non-movers, area-level absolute mobility now shows a significant negative association between area-level upwards mobility and abstention in the 2010 General Election. This is consistent with H1, although given the large number of parameters estimated across all models, we do not place much weight on this finding. As in the main results area-level relative mobility estimates are statistically non-significant across all outcomes.^13^

## Discussion

A well-established literature in political science shows that a ‘geography of discontent’ (McCann, 2020) has caused people in those areas left behind by the modern economy to feel politically alienated and to express that discontent through the ballot box. We have argued that one of the ways in which people feel let down is when a basic social contract is broken – people expect to do better than their parents and that their children will surpass their own achievements. Yet, whether, and how, living in a low social mobility area affects political alienation has been subject to surprisingly little empirical scrutiny. In this paper, we have considered this question using a novel dataset which has granular spatial estimates of social mobility derived from the UK decennial census based on 1% of the population. An additional contribution of our paper is that, for the first time, to our knowledge, we assess the relative contributions of relative and absolute measures of social mobility, an important distinction in the sociological literature on social mobility.

Our results show that beyond an individual’s own social mobility experience, lower historical levels of absolute social mobility in a local area were associated with a significantly higher probability of supporting ‘Leave’ in the 2016 European Union referendum. Living in a high mobility area (90th percentile) compared to a low mobility area (10th percentile) was associated with a 6.8 percentage points lower probability of supporting ‘Leave’. In substantive terms, this difference is large and would have been sufficient on its own to reverse the result. The findings for turnout in general elections were more mixed, with estimates in the expected direction and some statistically significant, but the overall pattern was of weak and predominantly non-significant effects. For the attitudinal measure of political efficacy, we find no support for the expectation that lower levels of social mobility in a local area diminishes the political efficacy of its residents.

Our findings also suggest, as expected, that the more relevant measure of social mobility is the absolute not the relative form, despite the latter being generally considered the more appropriate indicator of societal equality. While social fluidity – how our life outcome chances compare to those from different social class groups – may be a better measure of ‘fairness’, absolute mobility is more straightforward for individuals to observe, particularly at a local level. Individuals see how neighbours, colleagues, family members, and friends, who tend to live in close vicinity, fare according to this social contract of mobility.

As to why we find large effects of local area social mobility for Leave voting but not for general election turnout and political efficacy, we suggest both theoretical and methodological explanations. First, we argued that the EU Referendum was a clear opportunity to reject the status quo. The ‘Leave’ campaign strongly promoted the idea that the referendum was a battle between ordinary people and the political establishment (Hobolt, 2016). As Telford and Wistow (2020: 568) argued, the working class felt political abandoned the Labour Party and thus the referendum was a unique opportunity for those who felt resigned to their lack of control over the nation’s political economy to express their discontent. Abstention, on the other hand, can reflect a lack of political alternatives but could also derive from apathy rather than a rejection of the status quo. Regarding the null effects of low social mobility on political efficacy attitudes, here we think we are somewhat restricted by the questions available in *UKHLS*. This is to say that, because there are no direct measures of political alienation in the survey, we used political efficacy as a proxy for our target concept. We cannot conclude from this evidence alone, therefore, that area-level social mobility does not affect people’s sense of alienation from mainstream politics.

Methodologically, we must acknowledge that there is likely to be a high degree of random measurement error in our estimates of area-level social mobility. This is because we use the point estimates of relative and social mobility for each LAD derived by Buscha et al. (2021) from the ONS longitudinal study. Although the sample size of this study is very large (500,000 individuals at each wave), some of the confidence intervals of the area-level estimates are large, particularly for LADs with smaller populations. We have mitigated this somewhat by pooling the mobility estimates across cohorts, but it is likely that the residual noise will bias the coefficients for these key variables towards zero. In short, the true effects of social mobility on abstention, Brexit voting and political efficacy are likely to be larger than the estimates we have presented here. Thus, while we can rule out the possibility of large effects for the models where no statistically significant effects of social mobility were observed, we do not have sufficient power to reject the possibility of smaller effects that are real but close to zero. This is particularly relevant for the models of general election turnout where the pattern of findings was in the expected direction, but the results of the statistical tests were mixed.

A further limitation is the spatial unit at which we measure area-level social mobility, the LAD. LADs vary substantially in population size from approximately 25,000 to 1 million people and are larger than would ideally be the case for a mechanism based on local social interactions. Our choice was borne out of necessity, as the LAD is currently the lowest areal unit at which it is possible to produce robust measures of local social mobility in England and Wales. We must acknowledge, therefore, that part of the reason we have failed to find evidence of social mobility on our political alienation outcomes may be due to the spatial scale at which we have measured social mobility.

We tested our preferred models for robustness to alternative specifications (linear rather than logistic link function, treating area-level mobility as categorical rather than continuous), and for sub-groups (including/excluding those younger than 30 years old, including only non-movers, excluding the top and bottom decile of social mobility areas). With a small number of exceptions, these specifications are consistent with the main findings and support our overall conclusions. The area-level absolute mobility coefficient does become non-significant when we exclude the bottom and top deciles of the area social mobility distribution and when we include only ‘non-movers’. However, in both cases, the coefficient is of similar magnitude but measured less precisely due to a smaller sample size, so comparisons of statistical significance alone are not straightforward. And, while we mostly find null effects for H1 (higher area-level mobility is associated with less abstention), under some specifications, we find supporting evidence, so we cannot rule out a weak effect in some contexts for this outcome.

Our results have implications for the level of political alienation among future cohorts and how this is expressed electorally. It is widely acknowledged that most modern democracies are likely to experience a changing pattern of absolute mobility now and in the future. With substantial occupational upgrading over the later decades of the 20th century, most members of contemporary cohorts were born into middle-class families. From this higher base, it is inevitable that there will be a decline in upward social mobility with concomitant increases in downward mobility. These findings suggest that such a pattern is likely to have political consequences, such as was manifested in the anti-status quo character of the 2016 EU referendum.

## Supplemental Material

sj-docx-1-psx-10.1177_00323217241283930 – Supplemental material for Does Local Area Social Mobility Affect Political Alienation? Supplemental material, sj-docx-1-psx-10.1177_00323217241283930 for Does Local Area Social Mobility Affect Political Alienation? by Andrew McNeil and Patrick Sturgis in Political Studies

## Notes

1. Although interestingly, the opposite does not apply, that is, downwards mobility is not negatively associated with meritocracy, and upwards mobility not negatively associated with the structural explanation.

2. Based on NS-SEC 5 classifications, when occupational class was captured in the individual’s 40s.

3. UKIP did not win a seat and received 3.1% of the total vote share in the 2010 General Election. UKIP received 12.6% of the vote share in the 2015 General Election.

4. Turnout was 72.2% in the EU Referendum compared to 66.4% in the 2015 General Election.

5. This relationship between age and anti-system voting has recently come under question (Schäfer, 2021).

6. We include these response rates as an example, given our Brexit variable is from Wave 8 – all other response rates are available through UKHLS documentation, see https://www.understandingsociety.ac.uk/sites/default/files/downloads/documentation/user-guides/mainstage/responsetables.pdf

7. Buscha et al., subsumes NS-SEC 1 ‘large employers & higher management’ into NS-SEC 2 and 3.

8. We have interview dates to the month; therefore, there will be a very small number of individuals misclassified who responded between 23 and end of June 2016 who we have assumed were interviewed prior to the referendum.

9. Descriptive statistics are available in Supplementary Material Table 1.

10. We also include LAD-level controls for austerity and house price changes in Supplementary Material Table 23 for the Brexit model, which more closely matches the timing of our available data.

11. See Supplementary Material Figures 1 and 2 where we plot absolute mobility against relative mobility by LAD.

12. This ICC is not directly comparable with the ICC for the ‘Brexit’ and ‘abstention’ models estimated with a logit link function.

13. Given data limitations, the best match we can match is LAD to birth county. Thus, respondents potentially could have moved LADs within a county. Respondents may also have left their birthplace and later returned and still be counted as ‘non-movers’. In the Brexit model, 38% of individuals are ‘non-movers’ by this definition.
