# Supplementary material for: Does Local Area Social Mobility Affect Political Alienation?
Source: Polit Stud (Oxf). 2024 Oct 11;73(3):1106–25. doi: 10.1177/00323217241283930 (PMC12282958; doi:10.1177/00323217241283930)
Supplement: sj-docx-1-psx-10.1177_00323217241283930 – Supplemental material for Does Local Area Social Mobility Affect Political Alienation? [file sj-docx-1-psx-10.1177_00323217241283930.docx]

**Does local area social mobility affect political alienation?**

**Supplementary Material**

[**Table 1** Descriptive statistics –based on the with covariates Brexit model 2](#_Toc170812673)

[**Table 2** Multilevel Models, LAD level mobility and political alienation – Replacing relative odds ratio with long range relative odds ratio 3](#_Toc170812674)

[**Table 3** Correlation between mobility estimates and LAD level covariates 4](#_Toc170812675)

[**Table 4** Multilevel logistic models, abstention in the 2015 General Election (log odds) (Wave 7) 5](#_Toc170812676)

[**Table 5** Multilevel logistic models, abstention in the 2017 General Election (log odds) (Wave 9) 6](#_Toc170812677)

[**Table 6** Multilevel Models, LAD level mobility and voting intention in the next General Election Wave 3 (2011-2012) 7](#_Toc170812678)

[**Table 7** Multilevel Models, LAD level mobility and voting intention in the next General Election Wave 6 (2014-2015) 8](#_Toc170812679)

[**Table 8** Multilevel Models, LAD level mobility and voting intention in the next General Election Wave 9 (2017-2018) 9](#_Toc170812680)

[**Table 9** Multilevel linear probability models, LAD level mobility and ‘Leave’ support (Remain = 0, Leave = 1) 10](#_Toc170812681)

[**Table 10** Multilevel linear probability models, LAD level mobility and Abstention in the 2010 UK General Election (Voted = 0, Did not vote = 1) 11](#_Toc170812682)

[**Table 11** Multilevel logistic models predicting abstention in the 2010 General Election - Under 30-year-olds included and not restricting sample to those observations in the “Absolute & Relative & Covariates” version (log odds). 12](#_Toc170812683)

[**Table 12** Multilevel Logistic Models, LAD level mobility and ‘Leave’ Support - Under 30-year-olds included and not restricting sample to those observations in the “Absolute & Relative & Covariates” version (log odds). 13](#_Toc170812684)

[**Table 13** Multilevel Models, LAD level mobility and attitudes of political efficacy - Under 30-year-olds included and not restricting sample to those observations in the “Absolute & Relative & Covariates” version. 14](#_Toc170812685)

[**Table 14** Multilevel models full regression tables 16](#_Toc170812686)

[**Table 15** Multilevel Models - Excluding top and bottom decile absolute and relative mobility LADs. 18](#_Toc170812687)

[**Table 16** Multilevel models - Using quartiles of mobility ranges 19](#_Toc170812688)

[**Table 17** Multilevel models - including only individual level controls (no LAD level controls apart from mobility) 20](#_Toc170812689)

[**Table 18** Multilevel models - including only LAD level controls (no individual level controls) 21](#_Toc170812690)

[**Table 19** Unrotated factor analysis summary 22](#_Toc170812691)

[**Table 20** Rotated factor loadings 23](#_Toc170812692)

[**Table 21** - Multilevel models, political efficacy (Wave 3) by individual question 24](#_Toc170812693)

[**Table 22** Multilevel models - including only non-movers 25](#_Toc170812694)

[**Table 23** Multilevel models, including LAD level controls for change in LAD house prices and austerity 26](#_Toc170812695)

[**Figure 1** LAD Absolute upward mobility rates versus LAD long-range relative odds ratios 27](#_Toc170812696)

[**Figure 2** Absolute upward mobility rates versus LAD relative odds ratios 28](#_Toc170812697)

[**Figure 3** Predicted probabilities based on logistic multilevel models 29](#_Toc170812698)

**Table 1** Descriptive statistics –based on the with covariates Brexit model

|  | Mean | Std. Dev. |
| --- | --- | --- |
| LAD Level |  |  |
| Absolute Mobility | 20.3 | 2.7 |
| Relative odds ratio | 2.8 | 0.69 |
| Percent with degree | 27.2 | 7.21 |
| Percent not British | 8.64 | 7.32 |
| Median age | 40.9 | 4.94 |
| Percent white | 87.2 | 14.1 |
| Percent unemployed | 1.68 | 0.88 |
| GVA | 23581 | 9540 |
| Change in non-British born | 5.13 | 4.25 |
| Individual Level |  |  |
| Age | 48.3 | 10.3 |
| Sex |  |  |
| Male | 51.6% |  |
| Female | 58.4% |  |
| Race |  |  |
| White | 92.0% |  |
| Mixed | 1.1% |  |
| Asian | 4.5% |  |
| Other | 2.4% |  |
| Marital Status |  |  |
| Single | 20.5% |  |
| Married / cohabiting | 66.3% |  |
| Separated / Divorced | 11.5% |  |
| Widowed | 1.7% |  |
| Healthy |  |  |
| Long-term illness / disability | 27.8% |  |
| No long-term illness / disability | 72.2% |  |
| Income (monthly) | 2883 | 3259 |
| Highest Qualification |  |  |
| Degree | 37.1% |  |
| Other higher qualification | 13.6% |  |
| A-Level etc | 19.7% |  |
| GCSE etc | 19.2% |  |
| Other qualification | 7.4% |  |
| No quals | 3.0% |  |
| Social Mobility |  |  |
| Always high | 23.7% |  |
| Downwards | 14.3% |  |
| Upwards | 24.3% |  |
| Always low | 37.6% |  |
| Interviewed after ref | 71.6% |  |

Notes: The descriptive statistics are based on the sample from Model 10 in the main text. Data is weighted. The sample is somewhat restrictive as we include only those aged 30 and over, and working – so that we have an individual’s own social mobility and they are plausibly at their occupational ‘maturity’. We reproduce the models including under 30s in Supplementary Material Table 13.

**Table 2** Multilevel Models, LAD level mobility and political alienation – Replacing relative odds ratio with long range relative odds ratio

|  | Abstention UK GE 2010  (log odds) | Brexit  (log odds) | Political Efficacy  (Factor 1) | Political Efficacy  (Factor 2) |
| --- | --- | --- | --- | --- |
|  |  |  |  |  |
| **LAD Level** |  |  |  |  |
| Absolute Upward Mobility | -0.0241 | -0.0444* | -0.00186 | -0.000176 |
|  | (0.0256) | (0.0174) | (0.00371) | (0.00373) |
| Relative mobility – Long range | 0.0101 | 0.000129 | 0.00484 | -0.00003 |
|  | (0.0180) | (0.0127) | (0.00314) | (0.00340) |
| Other LAD level controls | Y | Y | Y | Y |
| Other individual level controls | Y | Y | Y | Y |
|  |  |  |  |  |
| Individuals | 5.604 | 8,503 | 13,334 | 13,334 |
| LADs | 316 | 329 | 330 | 330 |
| Random Effects |  |  |  |  |
| Variance (LAD) | 0.236 | 0.157 | 0.00638 | 0.00852 |
|  | (0.0552) | (0.0381) | (0.00146) | (0.00175) |
| Variance (individual) |  |  | 0.486 | 0.536 |
|  |  |  | (0.00612) | (0.00678) |
| Robust standard errors in parentheses | |  |  |  |
| *** p<0.001, ** p<0.01, * p<0.05 | |  |  |  |

Notes: Political efficacy based on Wave 3 of UKHLS as in the main results.

**Table 3** Correlation between mobility estimates and LAD level covariates

|  | Absolute mobility | Relative mobility |
| --- | --- | --- |
| Percent Degree | -0.1186 | 0.0857 |
| Not British | 0.3529 | -0.0869 |
| Percent white | -0.4325 | 0.1844 |
| Median age | -0.3597 | 0.0988 |
| Percent unemployed | 0.3955 | -0.0571 |
| GVA | 0.0132 | 0.0796 |
| Change in British born | 0.1823 | -0.0569 |

**Table 4** Multilevel logistic models, abstention in the 2015 General Election (log odds) (Wave 7)

|  |  |  |  |  |  |
| --- | --- | --- | --- | --- | --- |
|  | Empty | Absolute | Relative | Both | Absolute & Relative & Covariates |
|  |  |  |  |  |  |
| LAD absolute mobility |  | -0.00604 |  | -0.0132 | -0.00767 |
|  |  | (0.0231) |  | (0.0288) | (0.0357) |
| LAD relative mobility |  |  | -0.0392 | -0.0651 | -0.0594 |
|  |  |  | (0.0915) | (0.115) | (0.118) |
|  |  |  |  |  |  |
| Individual Level Controls | N | N | N | N | Y |
|  |  |  |  |  |  |
| LAD Level Controls | N | N | N | N | Y |
|  |  |  |  |  |  |
| Observations | 3,532 | 3,532 | 3.532 | 3,532 | 3,532 |
| Number of groups | 318 | 318 | 318 | 318 | 318 |
| Random Effects |  |  |  |  |  |
| Variance(LAD) | 0.418 | 0.415 | 0.419 | 0.415 | 0.300 |
|  | (0.0993) | (0.0998) | (0.0994) | (0.101) | (0.0915) |
| Robust standard errors in parentheses | |  |  |  |  |
| *** p<0.001, ** p<0.01, * p<0.05 | |  |  |  |  |

**Table 5** Multilevel logistic models, abstention in the 2017 General Election (log odds) (Wave 9)

|  | Empty | Absolute | Relative | Both | Absolute & Relative & Covariates |
| --- | --- | --- | --- | --- | --- |
|  |  |  |  |  |  |
| LAD absolute mobility |  | 0.00349 |  | -0.00868 | -0.0223 |
|  |  | (0.0223) |  | (0.0271) | (0.0333) |
| LAD relative mobility |  |  | -0.0893 | -0.108 | -0.120 |
|  |  |  | (0.0929) | (0.113) | (0.115) |
|  |  |  |  |  |  |
| Individual Level Controls | N | N | N | N | Y |
|  |  |  |  |  |  |
| LAD Level Controls | N | N | N | N | Y |
|  |  |  |  |  |  |
| Observations | 3,836 | 3,836 | 3,836 | 3,836 | 3,836 |
| Number of groups | 322 | 322 | 322 | 322 | 322 |
| Random Effects |  |  |  |  |  |
| Variance(LAD) | 0.372 | 0.372 | 0.372 | 0.373 | 0.267 |
|  | (0.120) | (0.120) | (0.119) | (0.120) | (0.0970) |
| Robust standard errors in parentheses | |  |  |  |  |
| *** p<0.001, ** p<0.01, * p<0.05 | |  |  |  |  |

**Table 6** Multilevel Models, LAD level mobility and voting intention in the next General Election Wave 3 (2011-2012)

|  | Empty | Absolute | Relative | Both | Absolute & Relative & Covariates |
| --- | --- | --- | --- | --- | --- |
|  |  |  |  |  |  |
| LAD absolute mobility |  | -0.0897 |  | -0.222 | -0.512** |
|  |  | (0.183) |  | (0.198) | (0.192) |
| LAD relative mobility |  |  | -0.589 | -0.984 | -1.000 |
|  |  |  | (0.607) | (0.670) | (0.607) |
|  |  |  |  |  |  |
| Individual Level Controls | N | N | N | N | Y |
|  |  |  |  |  |  |
| LAD Level Controls | N | N | N | N | Y |
|  |  |  |  |  |  |
| Observations | 11,748 | 11,748 | 11,748 | 11,748 | 11,748 |
| Number of groups | 330 | 330 | 330 | 330 | 330 |
| Random Effects |  |  |  |  |  |
| Variance(LAD) | 47.163 | 47.052 | 47 | 46.611 | 21.035 |
|  | (6.092) | (6.050) | (6.165) | (6.100) | (3.599) |
| Variance(individual) | 1167.123 | 1167.14 | 1167.106 | 1167.138 | 1060.065 |
|  | (23.402) | (23.400) | (23.403) | (23.397) | (20.477) |
| Robust standard errors in parentheses | |  |  |  |  |
| *** p<0.001, ** p<0.01, * p<0.05 | |  |  |  |  |

Notes: Voting intention is on a scale from 0-10, we have rescaled to a 0-100 scale. We have also flipped the scale, so 0 is “very likely” and 100 is “very unlikely” to make the interpretation clearly about abstention.

**Table 7** Multilevel Models, LAD level mobility and voting intention in the next General Election Wave 6 (2014-2015)

|  | Empty | Absolute | Relative | Both | Absolute & Relative & Covariates |
| --- | --- | --- | --- | --- | --- |
|  |  |  |  |  |  |
| LAD absolute mobility |  | -0.215 |  | -0.367 | -0.403 |
|  |  | (0.175) |  | (0.197) | (0.216) |
| LAD relative mobility |  |  | -0.471 | -1.152 | -0.921 |
|  |  |  | (0.561) | (0.640) | (0.633) |
|  |  |  |  |  |  |
| Individual Level Controls | N | N | N | N | Y |
|  |  |  |  |  |  |
| LAD Level Controls | N | N | N | N | Y |
|  |  |  |  |  |  |
| Observations | 10,376 | 10,376 | 10,376 | 10,376 | 10,376 |
| Number of groups | 330 | 330 | 330 | 330 | 330 |
| Random Effects |  |  |  |  |  |
| Variance(LAD) | 41.699 | 40.989 | 41.791 | 40.696 | 22.356 |
|  | (7.205) | (7.114) | (7.201) | (6.989) | (5.147) |
| Variance(individual) | 1068.061 | 1068.2 | 1067.972 | 1068.087 | 977.519 |
|  | (25.065) | (25.050) | (25.066) | (25.041) | (22.47) |
| Robust standard errors in parentheses | |  |  |  |  |
| *** p<0.001, ** p<0.01, * p<0.05 | |  |  |  |  |

Notes: Voting intention is on a scale from 0-10, we have rescaled to a 0-100 scale. We have also flipped the scale, so 0 is “very likely” and 100 is “very unlikely” to make the interpretation clearly about abstention.

**Table 8** Multilevel Models, LAD level mobility and voting intention in the next General Election Wave 9 (2017-2018)

|  | Empty | Absolute | Relative | Both | Absolute & Relative & Covariates |
| --- | --- | --- | --- | --- | --- |
|  |  |  |  |  |  |
| LAD absolute mobility |  | 0.112 |  | 0.124 | -0.0721 |
|  |  | (0.184) |  | (0.215) | (0.194) |
| LAD relative mobility |  |  | -0.139 | 0.0868 | -0.0877 |
|  |  |  | (0.645) | (0.760) | (0.652) |
|  |  |  |  |  |  |
| Individual Level Controls | N | N | N | N | Y |
|  |  |  |  |  |  |
| LAD Level Controls | N | N | N | N | Y |
|  |  |  |  |  |  |
| Observations | 7,334 | 7,334 | 7,334 | 7,334 | 7,334 |
| Number of groups | 330 | 330 | 330 | 330 | 330 |
| Random Effects |  |  |  |  |  |
| Variance(LAD) | 37.893 | 37.954 | 37.912 | 37.948 | 16.5 |
|  | (8.328) | (8.291) | (8.326) | (8.290) | (4.920) |
| Variance(individual) | 883.588 | 883.513 | 883.574 | 883.514 | 796.29 |
|  | (26.030) | (26.036) | (26.030) | (26.036) | (22.602) |
| Robust standard errors in parentheses | |  |  |  |  |
| *** p<0.001, ** p<0.01, * p<0.05 | |  |  |  |  |

Notes: Voting intention is on a scale from 0-10, we have rescaled to a 0-100 scale. We have also flipped the scale, so 0 is “very likely” and 100 is “very unlikely” to make the interpretation clearly about abstention.

**Table 9** Multilevel linear probability models, LAD level mobility and ‘Leave’ support (Remain = 0, Leave = 1)

|  | (6) | (7) | (8) | (9) | (10) |
| --- | --- | --- | --- | --- | --- |
|  | Empty | Absolute | Relative | Absolute & Relative | Absolute & Relative & covariates |
|  |  |  |  |  |  |
| LAD absolute mobility |  | -0.933** |  | -1.028* | -0.817* |
|  |  | (0.351) |  | (0.418) | (0.384) |
| LAD relative mobility |  |  | 1.093 | -0.720 | 0.317 |
|  |  |  | (1.120) | (1.374) | (1.085) |
| Individual social mobility (base always high) | |  |  |  |  |
| Downward |  |  |  |  | 8.410*** |
|  |  |  |  |  | (1.794) |
| Upward |  |  |  |  | 4.562** |
|  |  |  |  |  | (1.601) |
| Always low |  |  |  |  | 15.36*** |
|  |  |  |  |  | (1.733) |
|  |  |  |  |  |  |
| Individual Level Controls | N | N | N | N | Y |
|  |  |  |  |  |  |
| Lad Level Controls | N | N | N | N | Y |
|  |  |  |  |  |  |
| Observations | 8,503 | 8,503 | 8,503 | 8,503 | 8,503 |
| Number of groups | 329 | 329 | 329 | 329 | 329 |
| Random Effects |  |  |  |  |  |
| Variance (LAD) | 177.106 | 167.957 | 176.128 | 167.659 | 65.954 |
|  | (21.415) | (19.893) | (21.227) | (19.893) | (14.495) |
| Variance (individual) | 2296.474 | 2297.165 | 2296.555 | 2297.185 | 2054.63 |
|  | (23.899) | (23.859) | (23.893) | (23.860) | (26.824) |
| Robust standard errors in parentheses | |  |  |  |  |
| *** p<0.001, ** p<0.01, * p<0.05 | |  |  |  |  |

**Table 10** Multilevel linear probability models, LAD level mobility and Abstention in the 2010 UK General Election (Voted = 0, Did not vote = 1)

|  | (1) | (2) | (3) | (4) | (5) |
| --- | --- | --- | --- | --- | --- |
|  | Empty | Absolute | Relative | Absolute & Relative | Absolute & Relative & covariates |
|  |  |  |  |  |  |
| LAD absolute mobility |  | -0.0676 |  | -0.175 | -0.646 |
|  |  | (0.281) |  | (0.327) | (0.366) |
| LAD relative mobility |  |  | -0.544 | -0.880 | -1.669 |
|  |  |  | (1.071) | (1.252) | (1.172) |
| Individual social mobility (base always high) | |  |  |  |  |
| Downward |  |  |  |  | 5.694** |
|  |  |  |  |  | (1.961) |
| Upward |  |  |  |  | 3.837** |
|  |  |  |  |  | (1.181) |
| Always low |  |  |  |  | 7.288*** |
|  |  |  |  |  | (1.660) |
|  |  |  |  |  |  |
| Individual Level Controls | N | N | N | N | Y |
|  |  |  |  |  |  |
| Lad Level Controls | N | N | N | N | Y |
|  |  |  |  |  |  |
| Observations | 5,604 | 5,604 | 5,604 | 5,604 | 5,604 |
| Number of groups | 316 | 316 | 316 | 316 | 316 |
| Random Effects |  |  |  |  |  |
| Variance (LAD) | 68.996 | 68.768 | 68.871 | 68.304 | 41.27 |
|  | (14.062) | (13.990) | (14.140) | (13.958) | (9.620) |
| Variance (individual) | 1474.484 | 1474.56 | 1474.459 | 1474.64 | 1367.319 |
|  | (44.981) | (44.980) | (44.987) | (44.981) | (39.069) |
| Robust standard errors in parentheses | |  |  |  |  |
| *** p<0.001, ** p<0.01, * p<0.05 | |  |  |  |  |

**Table 11** Multilevel logistic models predicting abstention in the 2010 General Election - Under 30-year-olds included and not restricting sample to those observations in the “Absolute & Relative & Covariates” version (log odds).

|  | Empty | Absolute | Relative | Both | Absolute & Relative & covariates |
| --- | --- | --- | --- | --- | --- |
|  |  |  |  |  |  |
| LAD absolute mobility |  | 0.0226 |  | 0.0233 | -0.0488* |
|  |  | (0.0132) |  | (0.0150) | (0.0232) |
| LAD relative mobility |  |  | -0.0383 | 0.00539 | -0.0957 |
|  |  |  | (0.0423) | (0.0480) | (0.0767) |
|  |  |  |  |  |  |
| Individual Level Controls | N | N | N | N | Y |
|  |  |  |  |  |  |
| LAD Level Controls | N | N | N | N | Y |
|  |  |  |  |  |  |
| Observations | 17,795 | 14,750 | 14,750 | 14,750 | 6,914 |
| Number of groups | 397 | 337 | 337 | 337 | 317 |
| Random Effects |  |  |  |  |  |
| Variance(LAD) | 0.258 | 0.264 | 0.264 | 0.264 | 0.205 |
|  | (0.0382) | (0.0408) | (0.0413) | (0.0408) | (0.0455) |
| Robust standard errors in parentheses | |  |  |  |  |
| *** p<0.001, ** p<0.01, * p<0.05 | |  |  |  |  |

**Table 12** Multilevel Logistic Models, LAD level mobility and ‘Leave’ Support - Under 30-year-olds included and not restricting sample to those observations in the “Absolute & Relative & Covariates” version (log odds).

|  | Empty | Absolute | Relative | Both | Absolute & Relative & Covariates |
| --- | --- | --- | --- | --- | --- |
|  |  |  |  |  |  |
| LAD absolute mobility |  | -0.0307** |  | -0.0351* | -0.0492** |
|  |  | (0.0115) |  | (0.0145) | (0.0180) |
| LAD relative mobility |  |  | 0.0308 | -0.0316 | 0.0214 |
|  |  |  | (0.0363) | (0.0476) | (0.0568) |
|  |  |  |  |  |  |
| Individual Level Controls | N | N | N | N | Y |
|  |  |  |  |  |  |
| LAD Level Controls | N | N | N | N | Y |
|  |  |  |  |  |  |
| Observations | 25,635 | 20,988 | 20,988 | 20,988 | 9,709 |
| Number of groups | 390 | 338 | 338 | 338 | 329 |
| Random Effects |  |  |  |  |  |
| Variance(LAD) | 0.243 | 0.209 | 0.218 | 0.208 | 0.181 |
|  | (0.0236) | (0.0241) | (0.0259) | (0.0240) | (0.0376) |
| Robust standard errors in parentheses | |  |  |  |  |
| *** p<0.001, ** p<0.01, * p<0.05 | |  |  |  |  |

**Table 13** Multilevel Models, LAD level mobility and attitudes of political efficacy - Under 30-year-olds included and not restricting sample to those observations in the “Absolute & Relative & Covariates” version.

Factor 1

|  | Empty | Absolute | Relative | Both | Absolute & Relative & covariates |
| --- | --- | --- | --- | --- | --- |
|  |  |  |  |  |  |
| LAD absolute mobility |  | -0.00559 |  | -0.00567 | -0.00616 |
|  |  | (0.00332) |  | (0.00383) | (0.00361) |
| LAD relative mobility |  |  | 0.00958 | -0.000592 | -0.00926 |
|  |  |  | (0.0107) | (0.0121) | (0.0114) |
| Individual Level Controls | N | N | N | N | Y |
|  |  |  |  |  |  |
| LAD Level Controls | N | N | N | N | Y |
|  |  |  |  |  |  |
| Observations | 42,099 | 34,666 | 34,666 | 34,666 | 16,263 |
| Number of groups | 404 | 344 | 344 | 344 | 330 |
| Random Effects |  |  |  |  |  |
| Variance(LAD) | 0.0217 | 0.0183 | 0.0187 | 0.0183 | 0.00708 |
|  | (0.00243) | (0.00200) | (0.00205) | (0.00200) | (0.00134) |
| Variance(individual) | 0.611 | 0.612 | 0.612 | 0.612 | 0.495 |
|  | (0.00526) | (0.00581) | (0.00581) | (0.00581) | (0.00598) |
| Robust standard errors in parentheses | |  |  |  |  |
| *** p<0.001, ** p<0.01, * p<0.05 | |  |  |  |  |

Notes: Based on Wave 3 as in the main text.

Factor 2

|  | Empty | Absolute | Relative | Both | Absolute & Relative & Covariates |
| --- | --- | --- | --- | --- | --- |
|  |  |  |  |  |  |
| LAD absolute mobility |  | 0.00751* |  | 0.00776* | 0.00172 |
|  |  | (0.00296) |  | (0.00362) | (0.00390) |
| LAD relative mobility |  |  | -0.0121 | 0.00186 | -0.00792 |
|  |  |  | (0.0103) | (0.0127) | (0.0109) |
|  |  |  |  |  |  |
| Individual Level Controls | N | N | N | N | Y |
|  |  |  |  |  |  |
| LAD Level Controls | N | N | N | N | Y |
|  |  |  |  |  |  |
| Observations | 42,099 | 34,666 | 34,666 | 34,666 | 16,263 |
| Number of groups | 404 | 344 | 344 | 344 | 330 |
| Random Effects |  |  |  |  |  |
| Variance(LAD) | 0.0171 | 0.0170 | 0.0173 | 0.0170 | 0.00977 |
|  | (0.00181) | (0.00192) | (0.00196) | (0.00193) | (0.00175) |
| Variance(individual) | 0.575 | 0.575 | 0.575 | 0.575 | 0.533 |
|  | (0.00481) | (0.00515) | (0.00515) | (0.00515) | (0.00614) |
| Robust standard errors in parentheses | |  |  |  |  |
| *** p<0.001, ** p<0.01, * p<0.05 | |  |  |  |  |

Notes: Based on Wave 3 as in the main text.

**Table 14** Multilevel models full regression tables

|  | Abstain in 2010 UK GE  (log odds) | Brexit (log odds) | Political Efficacy (Factor 1) | Political Efficacy (Factor 2) |
| --- | --- | --- | --- | --- |
|  |  |  |  |  |
| LAD absolute mobility | -0.0444 | -0.0431* | -0.00397 | -0.00177 |
|  | (0.0274) | (0.0191) | (0.00384) | (0.00404) |
| LAD relative mobility | -0.124 | 0.00843 | -0.00197 | -0.0104 |
|  | (0.0915) | (0.0532) | (0.0128) | (0.0129) |
| Age | -0.0549*** | 0.00625* | -0.00905*** | 0.00196* |
|  | (0.00600) | (0.00318) | (0.000728) | (0.000785) |
| Female | -0.0936 | -0.298*** | 0.321*** | -0.0610*** |
|  | (0.0804) | (0.0519) | (0.0140) | (0.0143) |
| Race (white base) | |  |  |  |
| Mixed | 0.270 | -1.000*** | 0.0337 | 0.0102 |
|  | (0.419) | (0.301) | (0.0612) | (0.0698) |
| Asian | 0.290 | -0.131 | 0.153*** | 0.120*** |
|  | (0.263) | (0.133) | (0.0306) | (0.0310) |
| Other | 0.124 | -0.397* | 0.0323 | 0.0983* |
|  | (0.271) | (0.186) | (0.0427) | (0.0388) |
| Marital Status (single base) | | |  |  |
| Married | -0.329** | 0.0806 | 0.0356 | -0.0334 |
|  | (0.120) | (0.0752) | (0.0194) | (0.0232) |
| Separated | 0.354* | 0.260* | 0.0901*** | 0.0122 |
|  | (0.139) | (0.105) | (0.0241) | (0.0268) |
| Widowed | 0.373 | 0.361 | 0.126* | 0.0696 |
|  | (0.365) | (0.234) | (0.0526) | (0.0573) |
| No LT illness / disability | 0.0827 | -0.139* | 0.0275 | -0.0704*** |
|  | (0.106) | (0.0567) | (0.0157) | (0.0156) |
| Income | -5.26e-05 | -2.00e-05 | -5.75e-06 | -4.97e-07 |
|  | (3.45e-05) | (1.06e-05) | (3.05e-06) | (1.23e-06) |
| Qualification (degree base) | |  |  |  |
| Other higher degree | 0.516** | 0.765*** | 0.336*** | 0.136*** |
|  | (0.159) | (0.0910) | (0.0209) | (0.0233) |
| A-Level | 0.841*** | 0.938*** | 0.368*** | 0.199*** |
|  | (0.139) | (0.0763) | (0.0215) | (0.0248) |
| GCSE | 1.087*** | 1.114*** | 0.476*** | 0.254*** |
|  | (0.148) | (0.0843) | (0.0223) | (0.0230) |
| Other | 1.368*** | 1.174*** | 0.535*** | 0.316*** |
|  | (0.175) | (0.117) | (0.0265) | (0.0299) |
| No quals | 1.662*** | 1.404*** | 0.697*** | 0.292*** |
|  | (0.175) | (0.190) | (0.0350) | (0.0365) |
| Social mobility (base always high) | | |  |  |
| Downward | 0.492** | 0.428*** | 0.138*** | 0.119*** |
|  | (0.167) | (0.0900) | (0.0231) | (0.0231) |
| Upward | 0.401** | 0.259** | 0.0620** | 0.0724** |
|  | (0.123) | (0.0841) | (0.0192) | (0.0221) |
| Always low | 0.584*** | 0.730*** | 0.236*** | 0.202*** |
|  | (0.145) | (0.0847) | (0.0208) | (0.0227) |
| Interviewed after referendum |  | 0.130 |  |  |
|  |  | (0.0697) |  |  |
| LAD percent degree | -0.0116 | -0.0424*** | -0.00323 | -0.00711*** |
|  | (0.0121) | (0.00908) | (0.00169) | (0.00193) |
| LAD percent not British | 0.0139 | 0.0133 | -0.00395 | 0.000124 |
|  | (0.0165) | (0.0159) | (0.00280) | (0.00238) |
| LAD median age | 0.0155 | 0.0229 | 0.00393 | 0.00507 |
|  | (0.0178) | (0.0138) | (0.00262) | (0.00310) |
| LAD percent white | 0.00606 | -0.00749 | 0.00170 | -0.000462 |
|  | (0.00927) | (0.00665) | (0.00123) | (0.00128) |
| LAD percent unemployed | 0.180* | -0.0855 | -0.00312 | 0.0457** |
|  | (0.0749) | (0.0670) | (0.0135) | (0.0139) |
| LAD GVA | 5.65e-06 | 1.06e-07 | 1.44e-06 | 2.27e-06 |
|  | (8.12e-06) | (5.57e-06) | (1.14e-06) | (1.48e-06) |
| LAD change in Brit born | 0.000251 | -0.00675 | 0.00554 | 0.000312 |
|  | (0.0182) | (0.0133) | (0.00296) | (0.00304) |
| Constant | -0.166 | 0.256 | -1.046** | -0.386 |
|  | (1.514) | (1.085) | (0.345) | (0.211) |
|  |  |  |  |  |
| Observations | 5,604 | 8,503 | 13,334 | 13,334 |
| Number of groups | 316 | 329 | 330 | 330 |
| Random Effects | |  |  |  |
| Variance(LAD) | 0.23 | 0.157 | 0.00654 | 0.00851 |
|  | (0.0541) | (0.0381) | (0.00150) | (0.00174) |
| Variance(individual) | |  | 0.486 | 0.536 |
|  |  |  | (0.00612) | (0.00678) |
| Robust standard errors in parentheses | | |  |  |
| *** p<0.001, ** p<0.01, * p<0.05 | | |  |  |

**Table 15** Multilevel Models - Excluding top and bottom decile absolute and relative mobility LADs.

|  | Abstention UK GE 2010  (log odds) | Brexit  (log odds) | Political Efficacy (Factor 1) | Political Efficacy (Factor 2) |
| --- | --- | --- | --- | --- |
|  |  |  |  |  |
| **LAD Level** |  |  |  |  |
| Absolute Upward Mobility | -0.0320 | -0.0525 | -0.00861 | 0.00619 |
|  | (0.0344) | (0.0269) | (0.00567) | (0.00625) |
| Relative mobility | -0.250 | -0.0885 | 0.0111 | -0.0127 |
|  | (0.139) | (0.101) | (0.0207) | (0.0256) |
| Other LAD level controls | Y | Y | Y | Y |
| Other individual level controls | Y | Y | Y | Y |
|  |  |  |  |  |
| Individuals | 4,171 | 6,407 | 9,847 | 9,847 |
| LADs | 219 | 229 | 229 | 229 |
| Random Effects |  |  |  |  |
| Variance (LAD) | 0.175 | 0.116 | 0.00458 | 0.00863 |
|  | (0.0484) | (0.0367) | (0.00158) | (0.00196) |
| Variance (individual) |  |  | 0.488 | 0.537 |
|  |  |  | (0.00716) | (0.00770) |
| Robust standard errors in parentheses | |  |  |  |
| *** p<0.001, ** p<0.01, * p<0.05 | |  |  |  |

Notes: Political efficacy based on Wave 3 of UKHLS as in the main results.

**Table 16** Multilevel models - Using quartiles of mobility ranges

|  | Abstain in 2010 UK GE  (log odds) | Brexit  (log odds) | Political Efficacy  (Factor 1) | Political Efficacy  (Factor 2) |
| --- | --- | --- | --- | --- |
|  |  |  |  |  |
| LAD quartile of absolute mobility (base least mobile quartile) |  |  |  |  |
| 2nd quartile | -0.0930 | -0.124 | 0.00905 | 0.0123 |
|  | (0.148) | (0.0985) | (0.0210) | (0.0250) |
| 3rd quartile | -0.210 | -0.165 | 0.00280 | 0.0219 |
|  | (0.145) | (0.110) | (0.0241) | (0.0251) |
| 4th quartile (most mobile) | -0.133 | -0.324* | -0.0224 | 0.00707 |
|  | (0.167) | (0.148) | (0.0265) | (0.0294) |
| LAD quartile of relative mobility |  |  |  |  |
| 2nd quartile | 0.318* | -0.0124 | 0.0169 | -0.0321 |
|  | (0.134) | (0.111) | (0.0248) | (0.0260) |
| 3rd quartile | 0.223 | -0.0453 | 0.0397 | 0.00205 |
|  | (0.140) | (0.112) | (0.0250) | (0.0253) |
| 4th quartile (most fluid) | -0.0970 | -0.0207 | 0.0263 | -0.0122 |
|  | (0.149) | (0.117) | (0.0251) | (0.0288) |
|  |  |  |  |  |
| Individual Level Controls | Y | Y | Y | Y |
|  |  |  |  |  |
| LAD Level Controls | Y | Y | Y | Y |
|  |  |  |  |  |
| Observations | 5,604 | 8,503 | 13,334 | 13,334 |
| Number of groups | 316 | 329 | 330 | 330 |
| Random Effects |  |  |  |  |
| Variance(LAD) | 0.214 | 0.159 | 0.00626 | 0.00823 |
|  | (0.0489) | (0.0391) | (0.00148) | (0.00174) |
| Variance(individual) |  |  | 0.486 | 0.536 |
|  |  |  | (0.00612) | (0.00678) |
| Robust standard errors in parentheses |  |  |  |  |
| *** p<0.001, ** p<0.01, * p<0.05 |  |  |  |  |
|  |  |  |  |  |
|  |  |  |  |  |
|  |  |  |  |  |

**Table 17** Multilevel models - including only individual level controls (no LAD level controls apart from mobility)

|  | Abstention 2010 UK GE  (log odds) | Brexit  (log odds) | Political Efficacy (Factor 1) | Political Efficacy (Factor 2) |  |
| --- | --- | --- | --- | --- | --- |
|  |  |  |  |  |  |
| LAD absolute mobility | -0.0223 | -0.0351* | -0.0131*** | 0.00428 |  |
|  | (0.0235) | (0.0171) | (0.00356) | (0.00399) |  |
| LAD relative mobility | -0.0867 | -0.0150 | -0.00853 | -0.00498 |  |
|  | (0.0922) | (0.0548) | (0.0133) | (0.0151) |  |
|  |  |  |  |  |  |
| Individual Level Controls | Y | Y | Y | Y |  |
|  |  |  |  |  |  |
| LAD Level Controls | N | N | N | N |  |
|  |  |  |  |  |  |
| Observations | 5,604 | 8,503 | 13,334 | 13,334 |  |
| Number of groups | 316 | 329 | 330 | 330 |  |
| Random Effects |  |  |  |  |  |
| Variance(LAD) | 0.253 | 0.221 | 0.00955 | 0.0127 |  |
|  | (0.0577) | (0.0374) | (0.00185) | (0.00217) |  |
| Variance(individual) |  |  | 0.486 | 0.536 |  |
|  |  |  | (0.00611) | (0.00679) |  |
| Robust standard errors in parentheses | |  |  |  |  |
| *** p<0.001, ** p<0.01, * p<0.05 |  |  |  |  |  |
|  |  |  |  |  |  |
|  |  |  |  |  |  |
|  |  |  |  |  |  |
|  | | |  |  |  |
|  | | |  |  |  |

**Table 18** Multilevel models - including only LAD level controls (no individual level controls)

|  | Abstention 2010 UK GE  (log odds) | Brexit  (log odds) | Political Efficacy  (Factor 1) | Political Efficacy  (Factor 2) |  |
| --- | --- | --- | --- | --- | --- |
|  |  |  |  |  |  |
| LAD absolute mobility | -0.049 | -0.0447* | -0.00583 | -0.00264 |  |
|  | (0.0270) | (0.0184) | (0.00412) | (0.00406) |  |
| LAD relative mobility | -0.00413 | 0.00653 | -0.00329 | -0.0115 |  |
|  | (0.0180) | (0.0472) | (0.0153) | (0.00134) |  |
|  |  |  |  |  |  |
| Individual Level Controls | N | N | N | N |  |
|  |  |  |  |  |  |
| Lad Level Controls | Y | Y | Y | Y |  |
|  |  |  |  |  |  |
| Observations | 5,604 | 8,503 | 13,334 | 13,334 |  |
| Number of groups | 316 | 329 | 330 | 330 |  |
| Random Effects | |  |  |  |  |
| Variance(LAD) | 0.256 | 0.158 | 0.00844 | 0.00945 |  |
|  | (0.0569) | (0.0345) | (0.00187) | (0.00185) |  |
| Variance(individual) |  |  | 0.592 | 0.568 |  |
|  |  |  | (0.00782) | (0.00671) |  |
| Robust standard errors in parentheses | | |  |  |  |
| *** p<0.001, ** p<0.01, * p<0.05 | | |  |  |  |

**Table 19** Unrotated factor analysis summary

| Factor | Eigenvalue | Difference | Proportion | Cumulative |
| --- | --- | --- | --- | --- |
| Factor1 | 1.28203 | 0.57416 | 0.8408 | 0.8408 |
| Factor2 | 0.70788 | 0.93369 | 0.4643 | 1.3051 |
| Factor3 | 0.22581 | 0.01357 | -0.1481 | 1.157 |
| Factor4 | 0.23938 | . | -0.157 | 1 |

Notes: Factors derived from full sample in Wave 3 (N=44,915). Unrotated matrix.

**Table 20** Rotated factor loadings

| Variable | Factor1 | Factor2 | Uniqueness |
| --- | --- | --- | --- |
| Qualified to participate | 0.7163 | 0.1318 | 0.4696 |
| Better informed | 0.714 | 0.0655 | 0.4859 |
| Public officials don't care | 0.0694 | 0.6774 | 0.5364 |
| Say in government | 0.1385 | 0.6801 | 0.5183 |

Notes: Variables 3 and 4 are reverse coded. Rotated matrix: orthogonal.

**Table 21** - Multilevel models, political efficacy (Wave 3) by individual question

|  | Qualified | Informed | Officials | Say |  |
| --- | --- | --- | --- | --- | --- |
|  |  |  |  |  |  |
| LAD absolute mobility | -0.00491 | -0.00533 | -0.00554 | 0.000683 |  |
|  | (0.00490) | (0.00516) | (0.00489) | (0.00566) |  |
| LAD relative mobility | -0.00157 | -0.00632 | -0.0218 | -0.00319 |  |
|  | (0.0216) | (0.0126) | (0.0161) | (0.0160) |  |
|  |  |  |  |  |  |
| Individual Level Controls | Y | Y | Y | Y |  |
|  |  |  |  |  |  |
| Lad Level Controls | Y | Y | Y | Y |  |
|  |  |  |  |  |  |
| Observations | 13,388 | 13,394 | 13,376 | 13,384 |  |
| Number of groups | 330 | 330 | 330 | 330 |  |
| Random Effects | |  |  |  |  |
| Variance(LAD) | 0.0101 | 0.0113 | 0.0104 | 0.0156 |  |
|  | (0.00285) | (0.00226) | (0.00231) | (0.00324) |  |
| Variance(individual) | 0.974 | 0.763 | 0.891 | 0.990 |  |
|  | (0.0112) | (0.0102) | (0.0105) | (0.0107) |  |
| Robust standard errors in parentheses | | |  |  |  |
| *** p<0.001, ** p<0.01, * p<0.05 | | |  |  |  |

Qualified = I consider myself to be well qualified to participate in politics

Informed = I think I am better informed about politics than most people

Officials = Public officials don't care much about what people like me think (reverse coded)

Say = People like me don't have any say in what the government does (reverse coded)

**Table 22** Multilevel models - including only non-movers

|  | Abstention 2010 UK GE  (log odds) | Brexit  (log odds) | Political Efficacy  (Factor 1) | Political Efficacy  (Factor 2) |  |
| --- | --- | --- | --- | --- | --- |
|  |  |  |  |  |  |
| LAD absolute mobility | -0.112** | -0.0405 | -0.00957 | 0.000796 |  |
|  | (0.0422) | (0.00269) | (0.00599) | (0.00683) |  |
| LAD relative mobility | -0.0521 | 0.108 | -0.00633 | -0.0133 |  |
|  | (0.156) | (0.0893) | (0.0178) | (0.00213) |  |
|  |  |  |  |  |  |
| Individual Level Controls | Y | Y | Y | Y |  |
|  |  |  |  |  |  |
| Lad Level Controls | Y | Y | Y | Y |  |
|  |  |  |  |  |  |
| Observations | 1,873 | 3,229 | 5,255 | 5,255 |  |
| Number of groups | 276 | 314 | 326 | 326 |  |
| Random Effects | |  |  |  |  |
| Variance(LAD) | 0.362 | 0.199 | 0.00924 | 0.00928 |  |
|  | (0.131) | (0.0714) | (0.00329) | (0.00352) |  |
| Variance(individual) |  |  | 0.475 | 0.535 |  |
|  |  |  | (0.00936) | (0.0110) |  |
| Robust standard errors in parentheses | | |  |  |  |
| *** p<0.001, ** p<0.01, * p<0.05 | | |  |  |  |

Notes: Non-mover overestimates the number of individuals who do not move out of their LAD. Understanding Society’s lowest spatial unit for birthplace is county (the BHPS includes down to LAD level but then our sample would be much smaller as it would only include those respondents who were initiated in the BHPS). We include non-movers as anybody who lives in a LAD which is in the same county as their birth. Respondents may have moved LADs within a county and still be referred to as a “non-mover”. LADs are not perfectly nested in historic counties and thus we map each LAD to any possible county. Respondents may have also moved out of their birth LAD and returned by the wave in which the respective dependent variable is measured.

**Table 23** Multilevel models, including LAD level controls for change in LAD house prices and austerity

|  | | Brexit  (log odds) | |
| --- | --- | --- | --- |
|  | |  | |
| LAD absolute mobility | | -0.0427* | |
|  | | (0.0196) | |
| LAD relative mobility | | 0.00981 | |
|  | | (0.0550) | |
| Austerity | | -0.000843 | |
|  | | (0.000668) | |
| Change in House prices | | -0.0650 | |
|  | | (0.0798) | |
|  | |  | |
| Individual Level Controls | | Y | |
|  | |  | |
| Lad Level Controls | | Y | |
|  | |  | |
| Observations | | 8,498 | |
| Number of groups | | 328 | |
|  |  | |  |
| Variance(LAD) | | 0.150 | |
|  | | (0.0380) | |
|  | |  | |

Notes: Austerity measure taken from replication materials from Fetzer (2019), which in turn was based on Beatty and Feathergill (2013). Change in house prices are based on median LAD house percentage price change from 1996 to 2015 using replication data from Adler and Ansell (2020). Estimates for additional LAD variables are not available for 1 LAD hence the slight reduction in sample size.

**Figure 1** LAD Absolute upward mobility rates versus LAD long-range relative odds ratios

Notes: Long-range odds ratios are based on movements between NS-SEC 7-8 and NS-SEC 1-3

**Figure 2** Absolute upward mobility rates versus LAD relative odds ratios

**Figure 3** Predicted probabilities based on logistic multilevel models

Notes: Based on holding other covariates at their mean.

**Supplementary Materials References**

Adler, David, and Ben Ansell. 2020. ‘Housing and Populism’. *West European Politics* 43(2): 344–65. doi:10.1080/01402382.2019.1615322.

Beatty, Christina, and Steve Fothergill. 2013. *Hitting the Poorest Places Hardest: The Local and Regional Impact of Welfare Reform*. Sheffield Hallam University. doi:10.7190/cresr.2017.6378897426.

Fetzer, Thiemo. 2019. ‘Did Austerity Cause Brexit?’ *American Economic Review* 109(11): 3849–86. doi:10.1257/aer.20181164.
